# Angiomyolipoma in a Patient with Situs Inversus Totalis: Managing Two Rare Diseases

**DOI:** 10.1155/2016/5060284

**Published:** 2016-07-20

**Authors:** Jonathan Mayes, Nigel Heaton

**Affiliations:** ^1^Newcastle University, Newcastle upon Tyne NE1 7RU, UK; ^2^Institute of Liver Studies, King's Health Partners, King's College Hospital NHS FT, Denmark Hill, Camberwell, London SE5 9RS, UK

## Abstract

Hepatic angiomyolipoma is an extremely rare benign hamartomatous lesion. Situs inversus totalis is a genetic condition occurring in 0.01% of the population. Following the kidney, the liver is the second most common site of angiomyolipoma. No consensus on the treatment of hepatic angiomyolipoma has been reached. However, the majority of these tumours are managed conservatively. Situs inversus totalis presents difficulties for procedures and is most commonly an incidental finding. These two conditions have not previously been reported and no genetic link has been established between them. This paper reports the association of both conditions in a 74-year-old female, reviews the literature, and presents CT imaging of the case.

## 1. Background

Hepatic angiomyolipoma (AML) is a rare liver tumour composed of adipose tissue, smooth muscle cells, and blood vessels. Variations in composition give rise to varying radiological appearances [1]. Definitive pathological diagnosis is made with the identification of the three components and HMB-45 positive staining [2]. These tumours are considered to be benign and slow-growing lesions can be managed conservatively [3]. However, a small number have been noted to be malignant with evidence of recurrence after surgical resection [4, 5]. Angiomyolipoma occurs in 0.3% of the population, most commonly located in the kidney [1]. The liver is the second most common site, with approximately 300 cases being reported in the literature [6].

Situs inversus totalis is a rare autosomal recessive condition occurring in 0.01% of the population [7]. Currently, there are no known genetic conditions or variations connecting situs inversus with hepatic angiomyolipoma. The organs are mirrored throughout the body, the heart is placed in the right hemithorax, the left lung is trilobed, the right is bilobed, and liver lies on the left, whilst the stomach and spleen lie to the right. There is a 3–5% incidence of congenital heart disease, most commonly transposition of the great vessels. Clinical implications include unique anatomy for surgical interventions or procedure and expecting classical signs to be on the contralateral side.

Approximately 20% of patients also suffer from Kartagener syndrome, a congenital condition of ciliary dysfunction resulting in bronchiectasis and sinusitis [8].

## 2. Case Report

**Figure 1:**
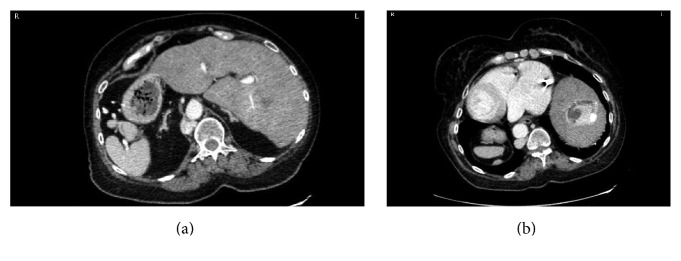
((a) and (b)) Transverse CT images of angiomyolipoma in a patient with situs inversus totalis.

**Figure 2:**
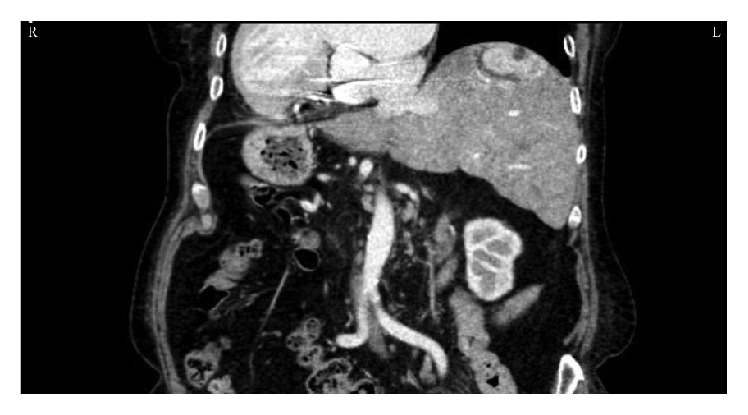
Coronal CT images of angiomyolipoma in a patient with situs inversus totalis.

A 74-year-old lady presented to accident and emergency department with vomiting and general malaise. She was found to have an acute kidney injury and metabolic acidosis. She had no history of liver disease. The past medical history included a cardiac pacemaker in situ for 20 years. Liver function tests were normal. Renal function was impaired with an eGFR of 38 mL/min. Tumour markers including serum alpha-fetoprotein, carcinoembryonic antigen, and carbohydrate antigen 19-9 levels were within normal limits. Due to a history of recent weight loss, abdominal CT scan was performed, demonstrating a large liver tumour and complete situs inversus (Figures 1 and 2).

The patient was considered unfit for surgery due to pulmonary hypertension and left atrial dilation/dysfunction. An echocardiogram revealed a dilated left atrium of 4.7 cm and an estimated left atrial volume of 68 mL. The decision was therefore undertaken to perform an ultrasound guided lesional biopsy. There was proliferation of epithelioid cells with an oncocytic appearance dispersed with an intervening florid lymphocytic reaction. Immunohistochemistry showed lesional cells staining for HMB-45 and smooth muscle actin. Hep-Par 1 and S100 stains were negative. Immunostaining for SAA was focal and proliferative activity was low, with Ki67 labeling less than 1% of neoplastic cells. The tumour was located in segment 8 in the right lobe of the liver and measured 61 × 57 × 46 mm.

These appearances were compatible with angiomyolipoma. Due to the low proliferation index and the high risk of surgery, a decision was made for conservative management. Follow-up was conducted with ultrasound at three-month intervals. After one year, there has been no change in morphology or lesion size.

## 3. Comment

Hepatic AML is an uncommon mesenchymal tumour composed of bloods vessels, smooth muscle cells, and adipose tissue in various proportions. Classically renal angiomyolipoma is associated with tuberous sclerosis [9]. Approximately 10% of cases of hepatic AML are associated with renal AML and tuberous sclerosis. It is typically echogenic on ultrasound, hypodense on precontrast CT scans, and markedly enhanced on arterial phase and remains moderately enhanced on the portal venous phase. MR imaging varies with the proportion of adipose tissue within the tumour [10]. The radiological images are often mistaken for hepatocellular carcinoma (HCC) and histological diagnosis is needed with HMB-45 immunostaining.

There is no consensus on AML management. Conservative management is recommended for asymptomatic patients who comply with follow-up, are not hepatitis virus carriers, and have tumours <5 cm in diameter. The diagnosis should be confirmed with fine needle aspiration or guided biopsy [11–13]. Surgery is infrequently needed to alleviate the mass effect on the neighbouring organs [1, 3]. There have been rare cases of hepatic AML rupturing, undergoing malignant transformation, and with concomitant hepatocellular carcinoma [4, 14, 15]. Situs inversus does not predispose to any liver disease but has implications for surgical management. Extremely rarely have hepatocellular carcinoma and situs inversus been reported and were they linked by liver cirrhosis associated with viral hepatitis [16–21].
